# 基于深度学习的血红蛋白等电聚焦电泳图谱快速识别分析

**DOI:** 10.3724/SP.J.1123.2024.05012

**Published:** 2025-06-08

**Authors:** Weichen JI, Youli TIAN, Haodong FU, Genhan ZHA, Chengxi CAO, Li WEI, Qiang ZHANG

**Affiliations:** 1.上海交通大学电子信息与电气工程学院，上海 200240; 1. School of Electronic Information & Electrical Engineering，Shanghai Jiao Tong University，Shanghai 200240，China; 2.海南师范大学化学化工学院，海南 海口 571158; 2. College of Chemistry and Chemical Engineering，Hainan Normal University，Haikou 571158，China; 3.上海交通大学附属第六人民医院，上海 200235; 3. Shanghai Sixth People’s Hospital，Shanghai Jiao Tong University，Shanghai 200235，China

**Keywords:** 深度学习, 条带检测, 无标志物, 等电聚焦电泳, 血红蛋白, deep learning, band detection, marker-free, isoelectric focusing （IEF） electrophoresis, hemoglobin （Hb）

## Abstract

基于轮廓分析的传统条带检测算法步骤繁琐，并且需要校正算法来应对背景不均匀、泳道扭曲和条带变形等问题。为了避免校正算法在检测过程中给条带分析结果所带来的误差，本文提出了一种基于深度学习目标检测算法的凝胶电泳图谱条带快速识别方法，并将该方法应用于血红蛋白（Hb）等电聚焦（IEF）电泳图谱的分析中。将通过微阵列IEF（mIEF）电泳实验收集的1 665张Hb IEF电泳图谱作为训练数据集，结合YOLOv8模型进行训练。依据模型推理得到的条带边界框位置和分类结果，对条带区域的像素灰度强度进行加和，以此计算各蛋白质的含量。研究结果表明，YOLOv8n模型在保持较低计算资源占用的同时，达到了92.9%的检测精度，实现了0.6 ms的推理时间，并成功应用于无等电点（pI）标志物条件下的Hb IEF电泳图谱条带的准确检测。以血红蛋白A2（Hb A2）为例，将本方法测得的Hb A2含量与临床检测结果进行对比，回归分析显示二者的线性度高达0.981 2，相关系数为0.980 0；进一步通过Bland-Altman分析法评估两种方法之间的一致性，结果表明，该方法与临床方法具有较好的一致性。与传统的自动条带检测方法相比，本文提出的方法快速、准确，重复性和稳定性更好。该方法可应用于临床实践中Hb A2含量的测定，并在成人*β*-地中海贫血疾病辅助诊断方面具备应用潜力。

## Abstract (en)

Gel electrophoresis is used to separate and analyze macromolecules （such as DNA， RNA， and proteins） and their fragments， and highly reproducible and efficient automatic band-detection methods have been developed to analyze gel images. Uneven background， low contrast， lane distortion， blurred band edges， and geometric deformation pose detection-accuracy challenges during automatic band detection. In order to address these issues， various correction algorithms have been proposed； however， these algorithms rely on researcher experience to adjust and optimize parameters based on image characteristics， which introduces human error while qualitatively and quantitatively processing bands. Isoelectric focusing （IEF） gel electrophoresis separates proteins with high-resolution based on isoelectric point （pI） differences. Microarray IEF （mIEF） is used for the auxiliary diagnosis of diabetes and adult *β*-thalassemia owing to operational ease， low sample consumption， and high throughout. This diagnostic method relies on accurately positioning and precisely determining protein bands. To avoid errors associated with correction algorithms during band analysis， this paper introduces a method for rapidly recognizing bands in gel electrophoresis patterns that relies on a deep learning object detection algorithm， and uses it to quantify and classify the IEF electrophoresis pattern of hemoglobin （Hb）. We used mIEF experiments to collect 1 665 pI-marker-free Hb IEF images as a model dataset to train the YOLOv8 model. The trained model accepts a Hb IEF image as input and infers band bounding boxes and classification results. Using inference data， the gray intensities of the pixels in each band area are summed to determine the content of each protein. The background and foreground of the image need to be separated prior to summing the abovementioned gray intensities， and the threshold method is used to achieve this. The threshold is defined as the average intensity of the background area， which is obtained by summing and averaging the background intensities of gel areas between the detection bounding boxes of each protein band. The baseline band areas are unified after removing the background. This method only requires the input image， directly outputs the corresponding electrophoretic band information， and does not rely on the experience of professionals nor is it affected by factors such as lane distortion or band deformation. In addition， the developed method does not depend on pI markers for qualitatively determining bands， thereby reducing experimental costs and improving detection efficiency. YOLOv8n delivered a detection accuracy of 92.9% and an inference time of 0.6 ms while using limited computing resources. Using Hb A2 as an example， we compared its content measured using the developed method with clinical data. The quantitative results were subjected to regression analysis， which delivered a linearity of 0.981 2 and a correlation coefficient of 0.980 0. We also used the Bland-Altman analysis method to verify that these two values are highly consistent. Compared with the traditional automatic band detection methods， the method developed in this study is fast， accurate， more repeatable， and stable， and can be used to determine the Hb A2 content in clinical practice， thereby potentially assisting in the auxiliary diagnosis of adult *β*-thalassemia.

凝胶电泳是一种可用于分离分析大分子（如DNA、RNA、蛋白质）以及大分子碎片的技术^［1］^。在电场作用下，不同尺寸大小或不同相对分子质量的生物分子会在泳道中形成条带^［2］^。为了提高凝胶图谱分析的重复性和效率，一些自动条带检测方法被提出^［3‒5］^，这些方法主要依赖于图像投影轮廓进行分析。然而，在自动条带检测过程中，不均匀的背景、低对比度、泳道扭曲、条带边缘模糊以及几何变形等问题，都对检测准确性构成了挑战。为了解决这些问题，已有多种校正算法被提出^［6‒9］^，但这些算法往往需要研究人员根据具体图像的特点进行参数调整和优化。这一过程不仅繁琐，而且高度依赖于专业经验，从而影响了条带分析结果的重复性。

作为一种凝胶电泳技术，等电聚焦（isoelectric focusing， IEF）电泳主要基于等电点（isoelectric point， pI）的差异来实现高分辨率的蛋白质分离^［10］^。微阵列IEF（microarray IEF， mIEF）电泳具有操作简便、样品消耗少和通量高等特点，可用于糖尿病^［11］^和成人*β*-地中海贫血疾病的辅助诊断^［12］^。这些诊断依赖于蛋白条带的精准定位和含量的精确测定。然而，在传统的IEF电泳图谱分析中，条带的定位通常依赖于专业人员的判断。在IEF过程中存在pH梯度漂移等问题，因此需要添加pI标志物来测算蛋白质的pI值^［13］^，但这一过程增加了材料成本和分析时间（单个样品分析耗时约10 min）。此外，IEF电泳条带可能会出现弯曲或形变的情况，这也增加了定量分析的难度。因此，为了降低IEF电泳图谱条带检测对专业人员的依赖，并提高分析效率和准确度，发展智能条带检测方法是必要的。

近年来，基于深度学习的视觉目标检测方法已被引入到生物医学图像分析中^［14］^，如定位超声扫描中的颈迷走神经^［15］^、基于Faster R-CNN自动检测和量化彗星检测图像中的DNA损伤^［16］^、无标签的免疫固定电泳分析^［17］^以及基于卷积神经网络的单细胞凝胶电泳分类^［18］^。深度学习算法有望实现IEF电泳图谱条带的快速检测。

在本文中，我们提出了一种基于YOLOv8模型的无pI标志物血红蛋白（hemoglobin， Hb）IEF电泳图谱条带检测方法，仅需输入图片，即可直接输出相应的电泳条带信息。该方法不依赖于专业人员的经验，也不受泳道扭曲和条带形变等因素干扰。此外，该方法对条带的定性不依赖于pI标志物，从而降低了实验成本，提高了检测效率。将训练后的模型用于血红蛋白A2（Hb A2）的含量测定，所得结果与临床检测数据展现出良好的一致性，表明所发展的方法具有一定的应用价值。

## 1 实验部分

### 1.1 仪器、试剂与材料

mIEF电泳成套设备（上海伯楷安生物科技有限公司）由电源（2 000 V）、聚焦托盘（87 mm×68 mm×12 mm）、动态扫描成像（dynamic scanning imaging， DSI）系统（检测波长405 nm）、传输系统、配备12根微分离柱（30 mm×600 μm×50 μm， pH 5.2~7.8）的mIEF阵列芯片（尺寸为72 mm×11 mm×8 mm）以及一个包含软件和硬件的完整聚焦系统组成。VARIANT Ⅱ血红蛋白测试系统（美国BIO-RAD公司）。

矿物油、氢氧化钠和磷酸均购自上海泰坦科技股份有限公司；载体两性电解质（pH 5.2~7.8）购自上海伯楷安生物科技有限公司；红细胞裂解液购自上海碧云天生物技术股份有限公司；血常规EDTA-K2抗凝管购自上药医疗器械有限公司。实验中用到的超纯水均由Milli-Q纯水仪（美国Millipore公司）制备。所有试剂均为分析纯。

### 1.2 样品前处理及mIEF电泳

研究使用上海交通大学附属第六人民医院采集的志愿者血样（包含两个地中海贫血病人血样），并已获得上海交通大学附属第六人民医院伦理委员会的批准（No：2019-036）。采集全血至抗凝管，使用红细胞裂解液将其稀释900倍，存放于‒20 ℃以供进一步使用。在进行mIEF分离前，先通过混合10 μL稀释血液、78 μL超纯水和 2 μL载体两性电解质（pH 5.2~7.8）来制备上样混合溶液；随后，取25 μL上样混合溶液于微分离柱中，放入mIEF阵列芯片，并在室温下孵育25 min以完成水化上样；将水化上样后的芯片放入电泳槽中，并加入4 mL矿物油以冷却并确保电泳过程稳定进行。电泳电压程序设定为初始200 V运行200 s，随后升压至800 V再运行800 s。电泳结束后，利用DSI系统在1 min内完成图像采集，并保存为8位灰度图像。

临床Hb A2含量的测定是通过VARIANT Ⅱ血红蛋白测试系统及其配套试剂，依据实验室标准操作规范来完成的^［19］^。本文所采用的临床验证数据由上海交通大学附属第六人民医院检测并提供。

### 1.3 条带检测模型构建

#### 1.3.1 模型数据集

针对Hb IEF电泳图谱条带分类的问题，我们建立了专属数据集，该数据集由1 665张IEF电泳图谱组成。这些IEF电泳图谱源自2022年3月至2023年6月期间，在上海第六人民医院收集的207个血样，每个血样经过5~7次的IEF电泳实验处理，且单次IEF电泳实验根据需求进行了单次或多次拍照，最终整理得到1 665张图片。这207个血样中包含205个Hb含量正常（即Hb A2在所有Hb中的含量占比<3.5%）样本，以及2个Hb含量异常（即Hb A2在所有Hb中的含量占比≥3.5%）样本。为了增加数据集中异常情况的占比，我们在实验操作和图像采集阶段采取了多种措施：搅动冷却液以产生气泡，人工手动调整分离柱的位置，以及手动调控光强和曝光时间，从而获得了不同光照和成像条件下的电泳图谱。使用LabelImg工具对IEF电泳图谱进行矩形边界标记（图1a），将图谱中的目标划分为6类，包括凝胶、气泡、糖化血红蛋白（Hb A1c）及血红蛋白A0（Hb A0）、高铁血红蛋白（MetHb）、胎儿血红蛋白（Hb F）和Hb A2。将数据集划分为训练集、测试集和验证集，其中1 290张图像用作训练集，199张图像用作测试集（以评估模型的最终性能），另有176张图像用作验证集（以在训练过程中验证模型效果，防止过拟合）。

**图1:**
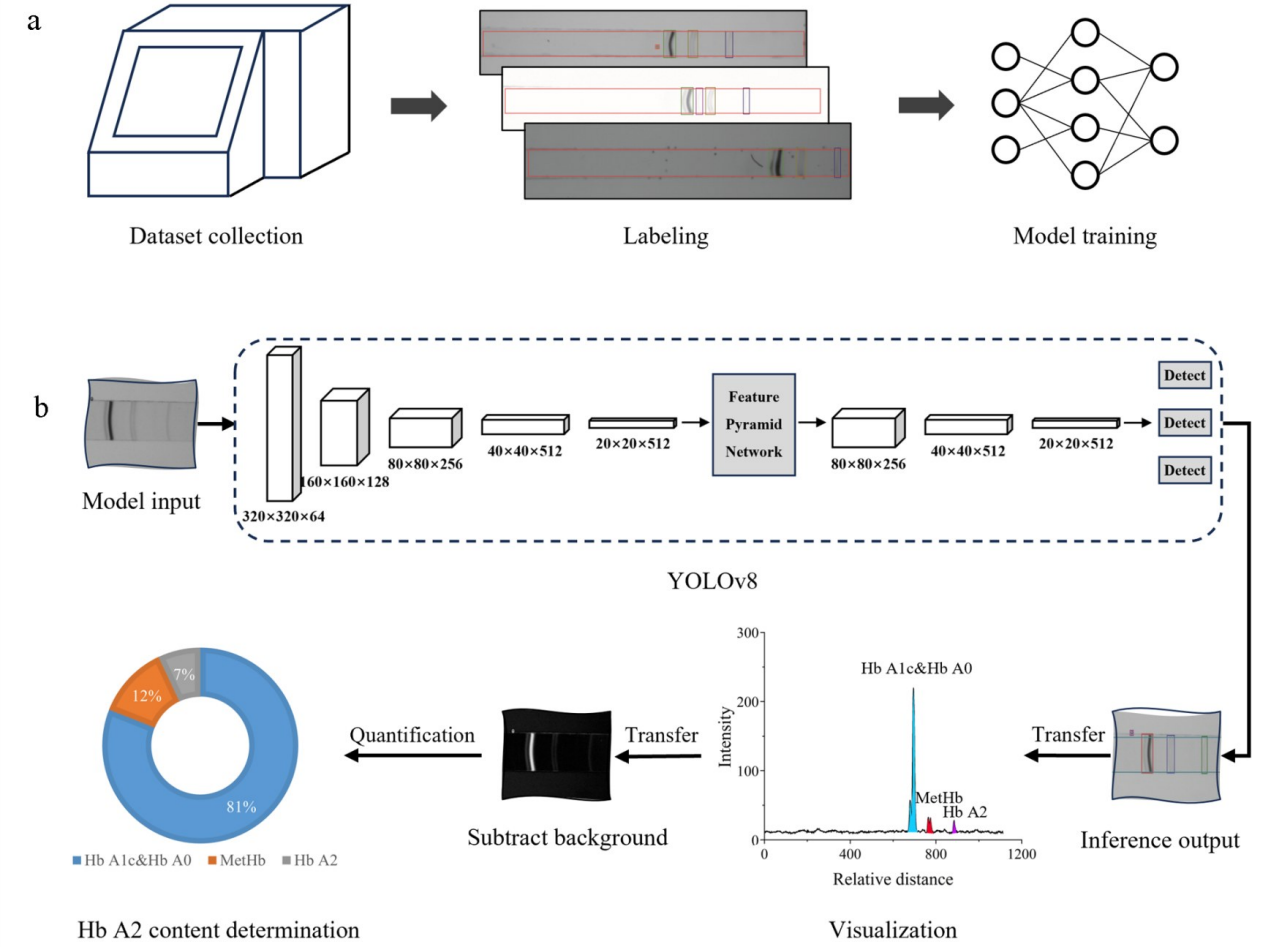
Hb IEF电泳图谱条带自动检测和定量方法流程

#### 1.3.2 模型训练

本文通过采用深度学习模型中的目标检测算法来解决电泳图谱中的条带定位和分类问题。在当前流行的目标识别方法及机器学习模型（如YOLO^［20］^（You Only Look Once）、Faster RCNN、SSD、RetinaNet）中，YOLO因其推理速度快、准确性和效率高而广受认可^［21］^。作为一种单阶段目标检测器^［22］^，YOLO将目标检测定义为一个单一的回归问题，能够直接从图像像素中预测出边界框坐标和类概率。YOLOv8是Ultralytics公司开发的YOLO物体检测和图像分割模型的最新版本，它在保持模型轻量化的同时，实现了更高检测准确度和更快速度的目标检测。如图1b所示，YOLOv8模型输入的是图像像素矩阵，经过轻量化的骨干网络（采用CSP-Darknet（Cross Stage Partial Darknet）结构）提取图像特征图，并利用多尺度特征融合技术将不同阶段的特征图进行融合，随后经过一系列的卷积层和池化层生成包含有预测框和类别的检测结果。通过控制模型的网络层数和输出特征图的通道数，YOLOv8提供了网络尺寸依次增大的推理变体，包括YOLOv8n（nano）、YOLOv8s（small）、YOLOv8m（medium）、YOLOv8l（large）和YOLOv8x（extra large）。其中YOLOv8n的参数最少，推理速度最快，适用于移动端部署；而YOLOv8x最为复杂，适用于对精度要求较高的场景。根据不同场景下的推理速度和精度需求，可以灵活选择合适的子模型。

YOLOv8模型的损失函数由分类损失（cls loss）和位置损失（bounding box loss）组成，其中cls loss采用交叉熵损失（binary cross-entropy loss， BCE loss）函数计算。bounding box loss由两部分组成，第一部分是计算预测框与目标框之间的交并比（intersection over union， IoU），采用完全交并比损失（complete intersection over union loss， CIoU loss）函数来计算；第二部分是分布式焦点损失（distribution focal loss， DFL loss）函数，它主要将框的位置建模为一个概率分布，使网络能够快速聚焦于与目标位置距离接近的分布。YOLOv8模型的总损失（Loss）由上述3类损失（BCE loss、CIoU loss和DFL loss）加权计算所得，计算公式如下：


Loss=BCE loss+7.5×CIoU loss+1.5×DFL loss （1）

在配备NVIDIA GeForce RTX 3090 GPU的计算机上，使用Pytorch（version 1.11.0）框架对该模型进行训练，将批次（batch）设置为16，这样可以充分利用硬件资源，同时确保训练时间保持在可接受范围内；将训练迭代次数（epochs）设置为30，在此设置下模型的收敛速度快，前5个周期内loss值迅速下降，并在25个周期左右保持稳定；根据数据集中图片的平均尺寸，将图片尺寸（image size）设置为640像素×640像素。

平均准确度（average precision，AP）是目标检测的标准性能度量^［23］^，而AP均值（mean AP，mAP）是将所有类别的AP相加后除以类别数得到的。在IoU阈值设定为0.5的条件下，将计算得到的mAP定义为mAP50。在性能评估方面，我们采用mAP50作为指标。

### 1.4 IEF电泳谱图条带检测和定量方法

Hb的IEF电泳图谱条带自动检测和定量方法流程如图1所示。首先，利用模型数据集对YOLOv8模型进行训练，随后将mIEF设备采集的Hb图谱作为模型输入；模型输出的推理结果包括条带检测边界框和Hb分类结果。基于这些推理结果，对分类后的蛋白条带进行含量计算。通过累加各条带检测框内像素的灰度值来获取条带强度，并将单个条带强度占所有检出条带合计强度的比例定义为该条带的含量。鉴于Hb A1c和Hb A0在图谱中的条带位置相近，为避免它们的检测框部分重叠导致在定量过程中重复计算背景强度，在标注和分类过程中将二者归为一类来处理。

在计算条带强度的过程中，如将检测框内所有像素的灰度值累加，会不可避免地计入背景强度，这将对条带的定量结果产生影响。因此，在图像灰度反转后，有必要采用背景去除算法来降低背景对条带含量计算的干扰，从而将条带周围的背景强度降至最低。本文采用阈值法对前、后景进行分离，将阈值定义为背景区域的平均强度。背景的平均强度是通过计算各蛋白条带检测框之间凝胶区域的背景强度总和后取平均值得到的。经过背景去除处理后，条带区域的基线基本达到了统一。观察图1b中展示的背景去除后的IEF电泳图谱，可见其条带区域相比原图泳道中的背景显得更加均匀。

## 2 结果与讨论

### 2.1 模型训练结果

从预测准确度、推理时间、参数量和GFLOPs（giga floating-point operations per second，用于衡量模型对计算资源的需求）4个方面对YOLOv8的5个子模型进行比较与优化。如图2a所示，YOLOv8的5个子模型（从n到x）在参数量和GFLOPs上呈现递增趋势，表明模型规模逐渐增大。由图2b可知，5个子模型（从n到x）的预测准确度依次为92.9%、93.4%、94.7%、94.6%和94.4%，前3个子模型的预测准确度随模型规模增大而升高，YOLOv8l和YOLOv8x模型的预测准确度略有下降；然而，更大的模型同时也意味着更大的计算开销，对应着更长的推理时间。其中，YOLOv8x模型的推理时间最长，约为6.2 ms，而YOLOv8n模型的推理时间最短，仅需0.6 ms。模型的推理时间与计算机硬件配置存在直接关系。在占用较少计算资源的基础上，YOLOv8n模型实现了可观的92.9%预测准确度。综合考虑以上因素，我们最终选定YOLOv8n作为应用模型。

**图2:**
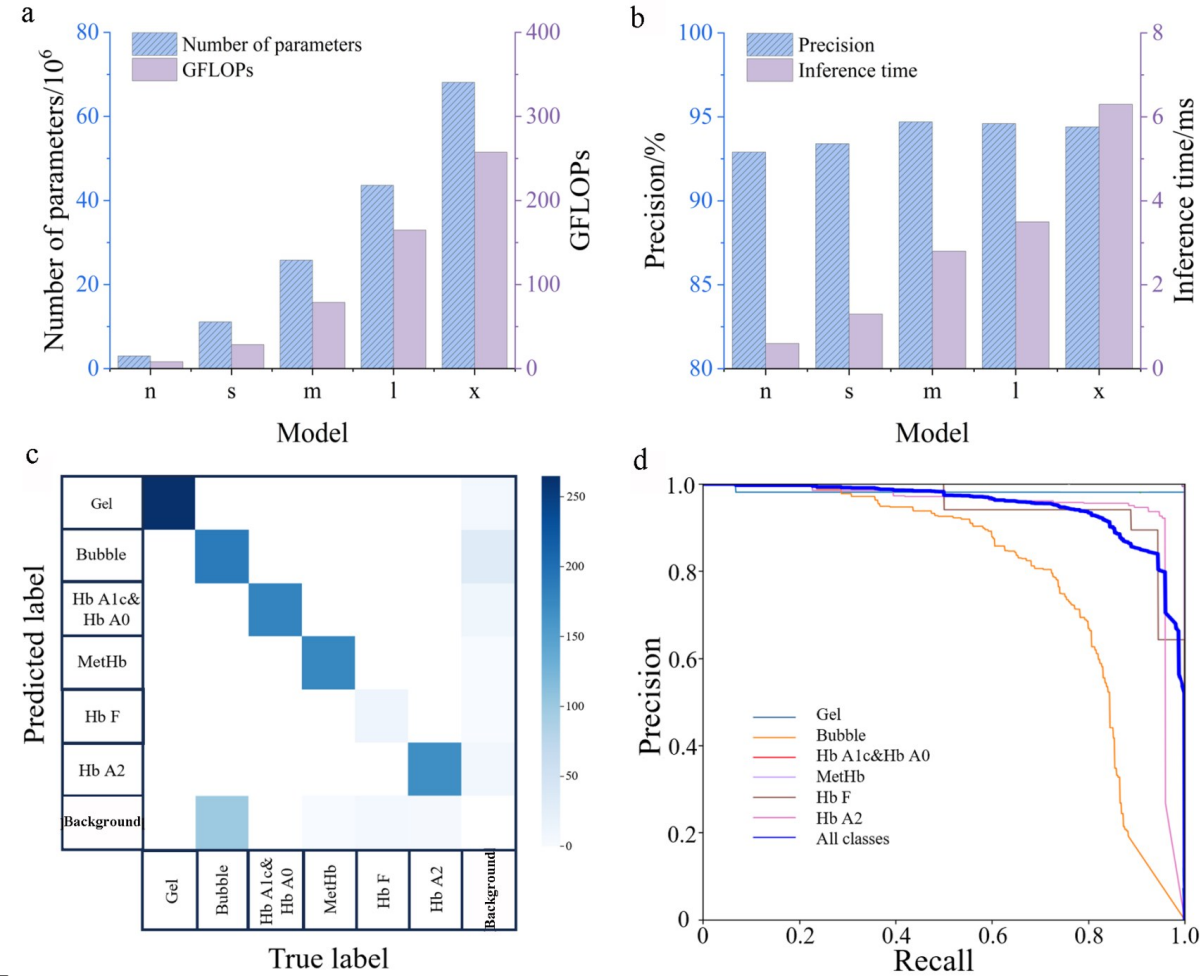
模型训练结果

图2c展示了YOLOv8n模型在训练结束后的预测混淆矩阵，该矩阵用于检验模型的分类表现。从图2c中可以看出，在6个类别中，凝胶和Hb A0&Hb A1c的识别准确度最高，均达到了100%；MetHb和Hb A2在预测过程中可能会被分类为背景，但整体误识别率较低，分别为3%和5%；相比之下，Hb F和气泡的识别准确度最低，分别为82%和74%。这可能是由于Hb F在模型数据集中的样本数量较少，同时气泡作为小目标难以被准确识别。因此，后续工作需要提高模型对Hb F的识别分类能力。图2d展示了YOLOv8n模型的准确度-召回率（precision-recall，PR）曲线，用于评估模型的性能。通过计算PR曲线的面积，可以得到各类别的mAP50值，其中凝胶、气泡、Hb A0&Hb A1c、MetHb、Hb F和Hb A2的mAP50分别为0.978、0.789、0.995、0.995、0.948和0.938。结果表明，本文所训练模型在条带识别方面表现良好，对各条带类别的预测准确率高且性能优异。

### 2.2 IEF电泳图谱分析流程对比

图3对比了基于轮廓法与YOLOv8模型方法的Hb IEF电泳图谱分析流程。如图3a所示，传统算法首先需要进行y轴投影，通过投影梯度等信息来定位IEF图谱中的电泳条带区域，以减少无关区域的影响；接着，进行背景校正，以减轻因光强不均一和背景不一致等问题所带来的影响；随后，对IEF图谱中的pI标志物进行定位；最终，实现信号峰的检测，并依据pI标志物的pI值和位置信息，对信号峰的pI进行计算和校正，完成峰的定性和含量计算。这一系列步骤较为繁琐，耗时较长，且可能存在误差积累的问题。相比之下，训练好的YOLOv8模型只需接收图片输入，即可直接输出最终的条带信息，实现从凝胶图像到分离分析结果的单步操作（图3b）。基于推理结果的条带分类和定量方法能够直接给出图谱中Hb的含量结果，显著减少了多步算法可能带来的误差积累，提升了分析效率。此外，传统算法高度依赖于pI标志物的添加、定位、图谱校正以及条带的定性，而本方法所采用的深度学习模型则实现了无pI标志物（marker-free）的条带定性和定位，进一步提高了分析效率。

**图3:**
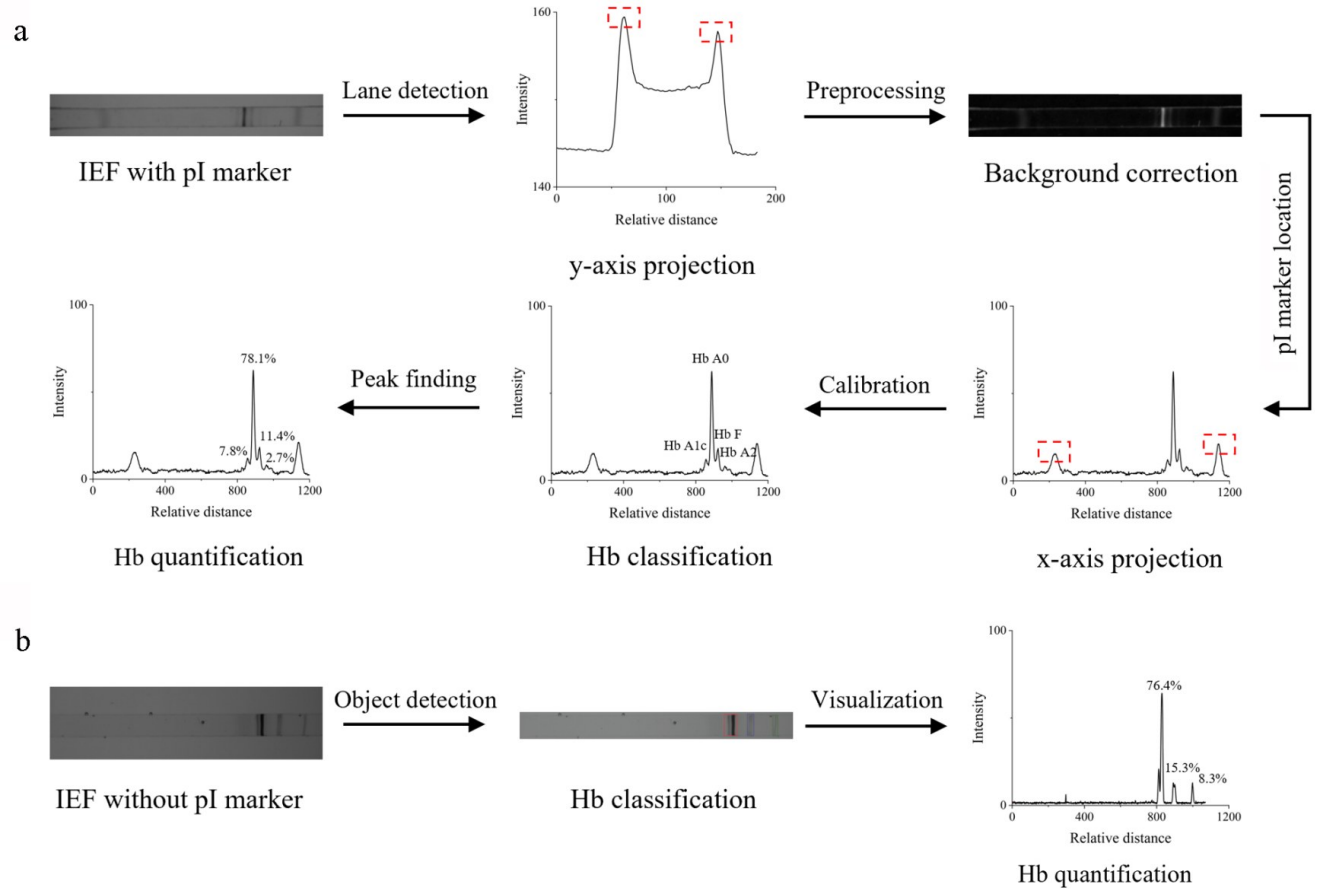
基于（a）轮廓法和（b）YOLOv8模型的Hb IEF电泳图谱分析流程对比

### 2.3 Hb IEF电泳图谱中Hb的含量测定

在IEF电泳凝胶成像过程中，曝光时间、泳道边缘、条带形状及其种类构成了实验所得IEF电泳图谱的特征。本研究针对IEF电泳图谱中Hb A2的含量进行了测定，并将测定结果与采用传统基于轮廓分析的自动检测算法所得定量结果进行了对比。图4a和4b分别展示了高曝光和正常曝光条件下存在特殊情况的IEF电泳图谱。对于图4a所示曝光时间较长的凝胶图谱，其背景更加均匀，但同时泳道信息难以读取。图4c对比了目标检测方法和轮廓分析方法对图4a的定量分析结果，结果显示，基于轮廓分析方法所测得的Hb A1c&Hb A0、Hb F、MetHb和Hb A2的含量分别为78.6%、2.1%、16.9%和2.4%，而基于深度学习目标检测方法得到的相应Hb含量分别为77.8%、3.6%、16.4%和2.2%。在无法划分泳道的情况下，轮廓分析方法可以基于x轴轮廓进行条带定位，但在计算条带区域时会将泳道区域外的背景算作条带，从而影响Hb条带含量的计算结果。

**图4:**
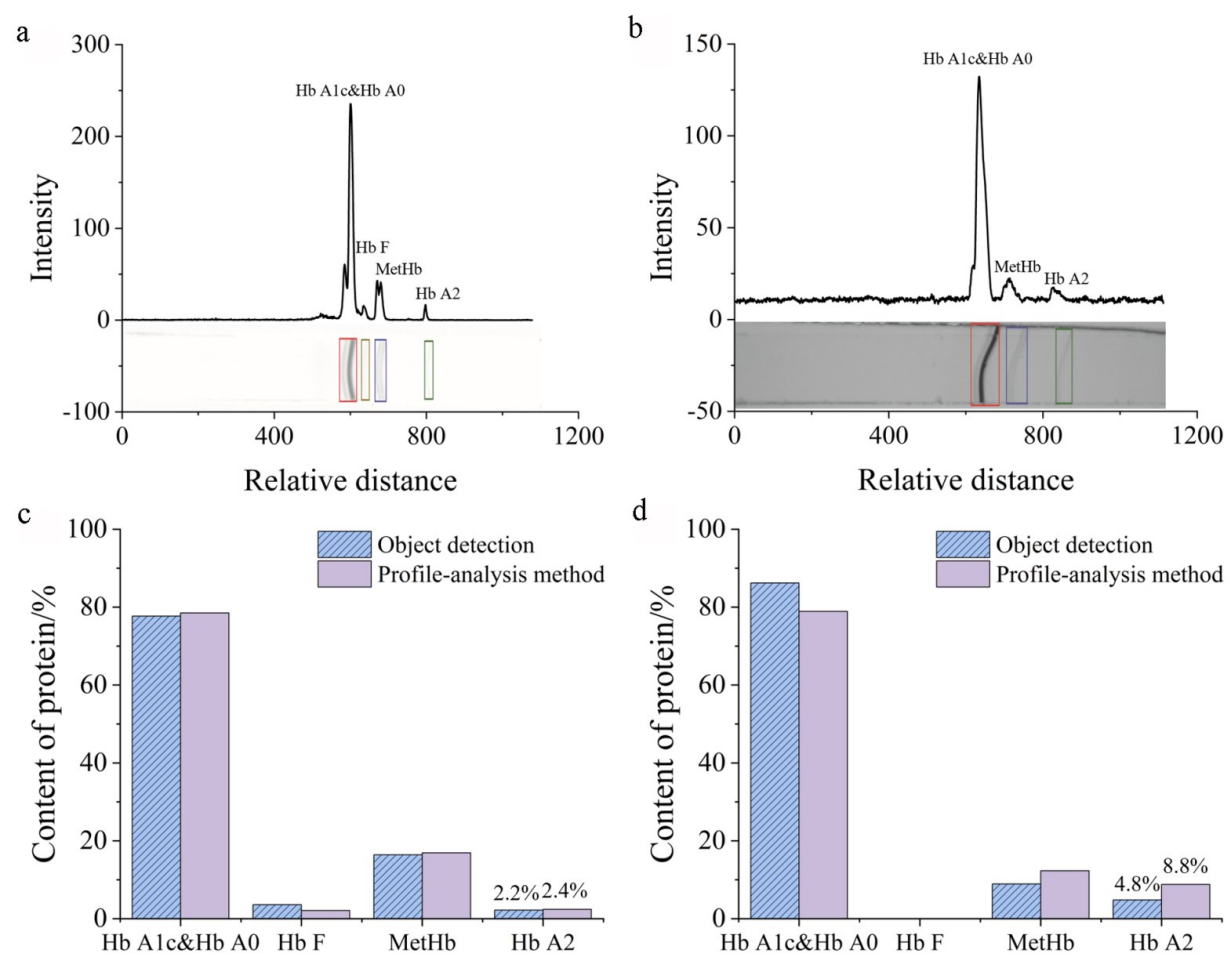
Hb IEF电泳图谱分析及mIEF条带含量测定结果

图4b展示的凝胶图像存在泳道边缘弯曲和条带形变的问题。因此，在采用轮廓分析方法进行条带定性之前，需先进行泳道矫正、背景校正以及条带矫正。图4d展示了通过两种方法测定的Hb含量结果：基于轮廓分析方法测得Hb A1c&Hb A0、Hb F、MetHb和Hb A2的含量分别为78.9%、0、12.3%和8.8%；而基于深度学习目标检测方法所得对应含量则分别为86.3%、0、8.9%和4.8%。在处理泳道扭曲、条带形变及低对比度条带时，基于轮廓分析的自动检测算法步骤较为繁琐，需依据经验调整参数，这不仅影响了定量结果的重复性，而且校正算法还会改变图谱区域的特征，进而对分析结果产生不利影响。

### 2.4 Hb A2含量测定结果的验证

为了验证本方法在含量测定方面的准确性，以Hb A2为例，将本方法测定结果与临床检测结果进行了对比。将本方法测定结果与临床检测结果进行线性回归分析，所得到的线性度为0.981 2，相关系数为0.980 0（图5a），这证明了基于深度学习目标检测的mIEF电泳图谱条带识别定量方法与临床检测结果一致。进一步地，利用Bland-Altman分析法评估了两种方法的一致性（图5b），结果显示，本方法的计算值与临床结果的差值均数（Mean）为0.003 6%（由红色线表示），一致性界限（‒1.96SD~1.96SD）为‒0.078 9%~0.086 1%，且差值均位于95%的一致性界限内，表明本方法与临床方法在测定Hb A2含量时具有较好的一致性。因此，该方法可应用于临床实践中Hb A2含量的测定。

**图5:**
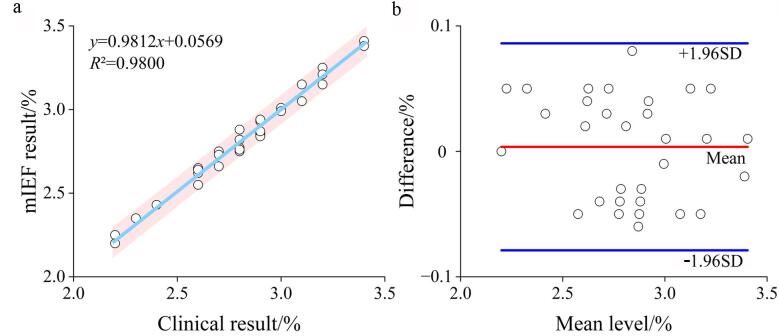
Hb A2的含量测定结果对比

本方法旨在实现IEF电泳图谱中条带的精确识别和定量。准确测定临床样本中的Hb A2含量对于辅助成人*β*-地中海贫血的疾病诊断具有重要意义。然而，上海地区成人*β*-地中海贫血的患病率低于1%，导致数据集中缺少该疾病的样本。实验室目前正在广西南宁地区收集成人*β*-地中海贫血的血样，并计划在后续工作中增加数据集中该疾病IEF图谱的比例。

## 3 结论

本文提出了一种基于YOLOv8模型的Hb IEF电泳图谱条带识别与分析方法，可用于Hb A2的含量测定，提高了Hb IEF电泳图谱的分析效率。实验结果表明，所训练的模型对mIEF电泳图谱条带检测的准确度高达92.9%，与传统条带检测方法相比，本方法基于目标检测原理，能够更快速且准确地分析mIEF电泳图谱。在Hb A2含量测定方面，本方法与临床检测数据展现出良好的一致性，显示出在成人*β-*地中海贫血疾病辅助诊断中的应用潜力。然而，该方法在面对位置相近且可能存在弯曲情况的两种蛋白条带时，其检测与分类能力存在局限。因此，后续工作计划采用基于深度学习的图像分割技术，以实现此类复杂情况下的蛋白条带分类检测与含量测定，从而进一步提升分析准确度。
